# Evaluation of the injured runner: developing the clinical hypothesis

**DOI:** 10.1186/1757-1146-4-S1-A4

**Published:** 2011-05-20

**Authors:** Irene Davis

**Affiliations:** 1 Dept. of Physical Medicine and Rehabilitation, Harvard Medical School, Cambridge, MA, 02138, USA

## 

The etiology of running injuries is multifactorial in nature. These factors can be simplified into those related to structure, mechanics and dosage. In order to fully understand the cause of an injury, one must carefully assess each of these factors. This includes taking a thorough history, as well as an evaluation of runner’s structure and alignment. However, if the injury occurred during the running, then the runner should also be assessed while running. With information on structure, running mechanics and dosage, one can then develop a clinical hypothesis upon which the treatment is then based. This presentation will review the important components of an evaluation of an injured runner, as well as the development of the clinical hypothesis. The presentation will end with a case study, which will reinforce these concepts.

